# Silvio Weidmann: laying the foundations for unravelling the mechanism of heart rhythm

**DOI:** 10.1098/rstb.2022.0161

**Published:** 2023-06-19

**Authors:** Dario DiFrancesco, Denis Noble

**Affiliations:** ^1^ Department of Biosciences—The PaceLab, University of Milan, via Celoria 26, 20133 Milan, Italy; ^2^ Balliol College, University of Oxford, Oxford OX1 3BJ, UK; ^3^ Department of Physiology, Anatomy and Genetics, University of Oxford, Oxford OX1 3PT, UK

**Keywords:** history of cardiac electrophysiology, sodium current, AP modelling, conductance measurements, *G*_K_-decay theory, funny current

## Abstract

Silvio Weidmann laid the basis of cardiac electrophysiology and was the forerunner in the search for mechanisms governing the electrical activity of the heart in his legendary first studies of Purkinje fibres in the 1950s. His work was the cornerstone of research in this field for many generations, and countless cardiologists and electrophysiologists have based their studies on the knowledge generated by Weidmann's pioneering data. This review summarizes his key contributions from the first intracellular recordings of cardiac membrane potentials in 1949 to the publication of his monograph in 1956. That summary is followed by an imagined dialogue between the authors and Silvio Weidmann himself, in the format of a one-act play. Both of us have such good recollections of our real-life conversations with Silvio Weidmann that we decided we could achieve a better feel for the history and issues by using a dialogue format. We hope that, in that way, we may transmit the character of Silvio Weidmann better for those readers who will not have known him personally. Silvio Weidmann was an extraordinarily sensitive and conversational person as well as a great scientist, and we feel it is worth the effort to convey that fact here.

This article is part of the theme issue ‘The heartbeat: its molecular basis and physiological mechanisms’.

## 1. Introduction

**Figure 1.**
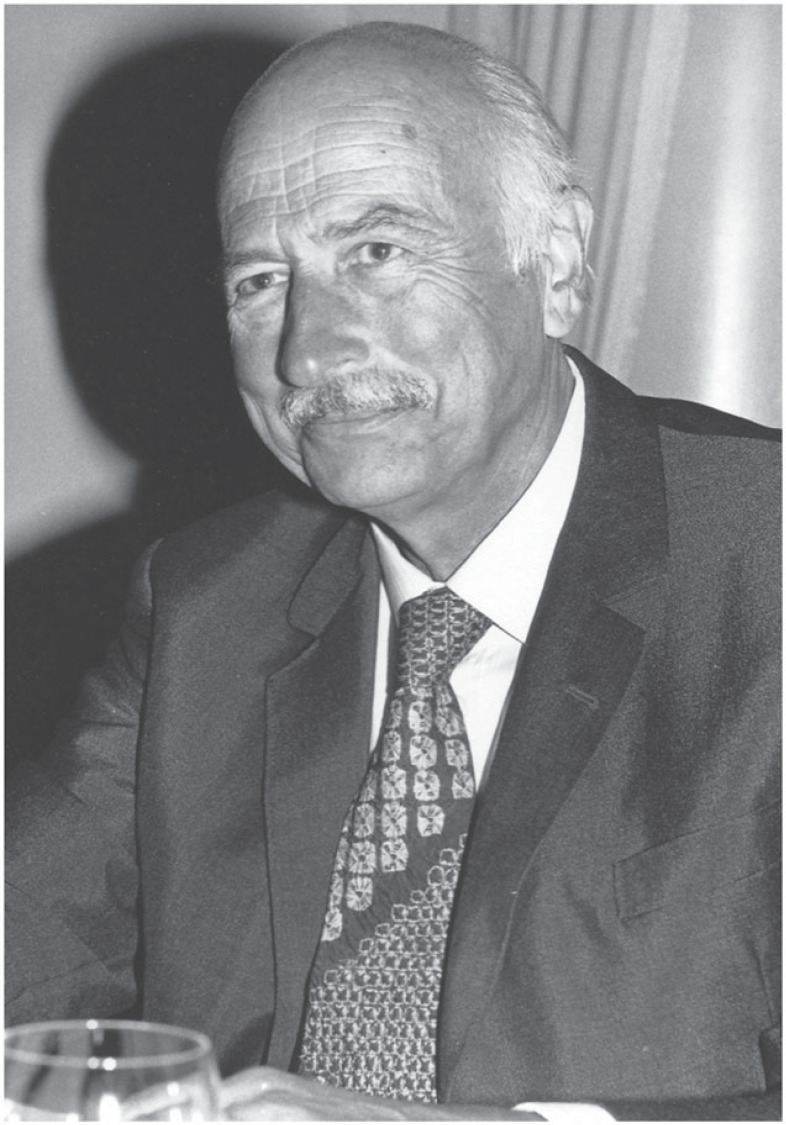
Silvio Weidmann at the farewell dinner of the 1986 Silvio Weidmann Symposium in Bern. Courtesy Ernst Niggli.

The Swiss cardiac electrophysiologist Silvio Weidmann (figure 1) was born in 1921, just over a century ago. He passed away in 2005 at the age of 84. This issue of *Philosophical Transactions B* on ‘The heart rhythm: its molecular basis and physiological mechanisms' almost coincides with the centenary of his birth. In prefacing this theme issue, our article is therefore devoted to celebrating his life and his role as the founding pioneer of electrophysiology of the heart.

## 2. Life

Silvio Weidmann was born in Bern in April 1921. He studied Medicine in Bern and obtained his MD in 1947, after which he moved to the University of Uppsala to specialize in Physiology. In 1948, he went to Cambridge with a postdoctoral fellowship and joined the Physiological Laboratory, working in Alan L. Hodgkin and Andrew F. Huxley's laboratory at a time when the fundamentals of membrane electricity were being unveiled with the development of the voltage-clamp technique to investigate the nerve action potential. During this time, he learned how to pull a microelectrode and impale cardiac cells, and published with Edouard Coraboeuf the first recording of a cardiac action potential. After returning to Bern in 1950, inspired by what he had learned in Cambridge, he refined the newly acquired technique and eventually developed a voltage clamp for Purkinje fibres. His contributions to technical innovations and his seminal studies opened a new field of research to investigate the electrical properties of the heart. The laboratory in Bern became for years to come a leading laboratory for researchers entering the field of cardiac electrophysiology and attracted countless visitors.

Progress in his academic career led Silvio Weidmann to first became Assistant (1951), then Associate (1955), then Full Professor (1958) at the University of Bern. He also went abroad, and spent a year at the State University of New York (SUNY) Downstate Medical School in 1954 as a Visiting Professor. He became Head of the Bern Institute of Physiology in 1968–1986, and Chancellor of the University of Bern in 1974–1975.

Universally recognized as a pioneer in the field of cardiac cellular electrophysiology, he received several awards, among which were the Theodor Kocher Prize in 1957 and Honoris Causa doctoral degrees from the universities of Paris-Sud in 1976, Uppsala in 1977 and Leicester in 1982.

## 3. Basic experimental findings

Weidmann was the first, in 1949, to record membrane potentials in cardiac Purkinje fibres using intracellular glass microelectrodes [1] to replace the inaccurate method of external suction electrodes. That work, published in French, established that the resting potential is negative to −70 mV, but it failed to record the rapid overshoot, probably because the input resistance of the recording amplifier was too low. That problem was overcome when Weidmann moved to Cambridge to work with M. H. Draper in Alan Hodgkin's laboratory, where they showed that the overshoot rapidly reaches between +20 and +40 mV and that the resting potential averages −90 mV. Using fast sweep oscilloscope recordings, they also measured the speed of the upstroke [2]. This work required high impedance cathode followers to which the recording electrode could be connected.

Weidmann then worked alone to rapidly establish the founding observations of cardiac electrophysiology. He recorded the time course of electronic potentials and their exponential decay as a function of distance from the polarizing electrode [3]. This work required two microelectrodes to be inserted into the Purkinje fibres at various distances from each other, and the results enabled estimates of the membrane and intracellular resistances and membrane capacitance required for any simulation of conduction in cardiac tissue.

The two microelectrode technique was then used in a remarkable experiment using repetitive current pulses and superimposition of successive action potentials to demonstrate the time course of membrane resistance changes throughout the cardiac cycle.

**Figure 2.**
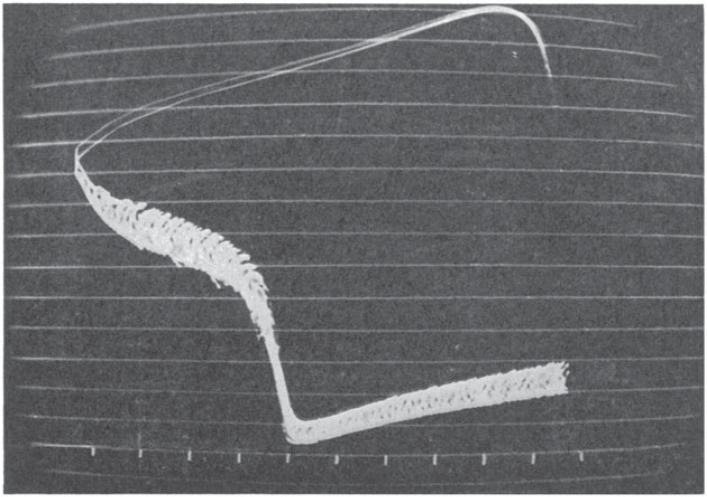
Oscilloscope recordings of membrane resistance changes in a Purkinje fibre during the cardiac cycle. Repetitive square pulses of current were injected through one microelectrode while the membrane potential and its repetitive displacements were recorded with the second microelectrode. Horizontal lines are 10 mV apart, while the time marks are at 100 ms intervals [4].

That iconic oscilloscope image (figure 2) became the target of later computer simulations of cardiac action potentials. It showed the key features that were later to become so important in debates on the ionic mechanisms involved at each stage of the cycle:

(1) the resistance is very low indeed during the fast upstroke of the action potential, consistent with a large increase in conductance. The demonstration that the overshoot behaved like a sodium electrode confirmed that the upstroke is caused by a large increase in permeability to sodium ions.
(2) the resistance then rapidly recovers and reaches its peak during the later part of the action potential plateau;
(3) it falls again somewhat during the repolarization phase; and
(4) it shows a gradual decline during the pacemaker depolarization [4]. This result was initially interpreted in the *G*_K_-decay theory to imply a declining potassium conductance as the basis of pacemaker rhythm.

**Figure 3.**
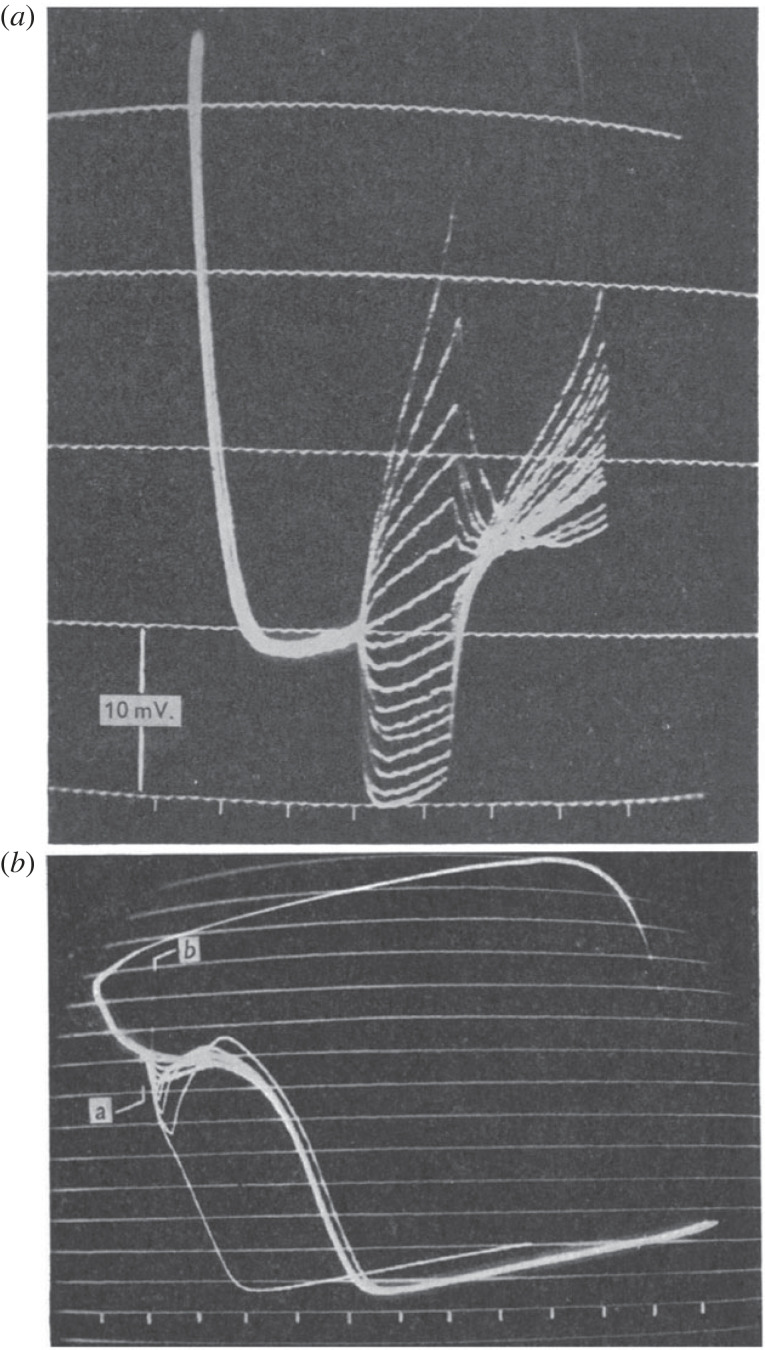
The nonlinear responses to larger current pulses. (*a*) Pulses of various magnitudes applied during the pacemaker depolarization showing a threshold for excitation of an action potential. (*b*) Pulses of various amplitudes applied during the action potential plateau showing a threshold for depolarization [4].

It is important to note that the magnitude of the current pulses used in this experiment was carefully adjusted to remain in a range within which the membrane could be interpreted to behave as an ohmic resistor. When Weidmann applied much larger pulses, he discovered the existence of all-or-nothing depolarization, showing that the membrane properties are highly nonlinear (figure 3).

All these experiments were subsequently published in German in Weidmann's famous monograph *Elektrophysiogie der Herzmuskelfaser* [5]. That monograph became a treasured source for the early development of mathematical models of cardiac action and pacemaker potentials.

Initially, it was thought that the interpretation of Weidmann's experiments was straightforward since the first mathematical simulations [6,7] succeeded in fully reconstructing all the key experimental findings in that monograph. It was not until the late 1970s that it became clear that the situation is much more complex. By that time, calcium channels and the sodium–calcium exchange had been identified by Harold Reuter [8], while the *G*_K_-decay theory for the pacemaker potential was challenged by the discovery of the ‘funny’ hyperpolarizing-activated mixed cation channel [9], followed by the re-interpretation of the Purkinje fibre's *I*_K2_ current [10,11] and the re-modelling of the electrical activity of Purkinje fibres [12].

Weidmann maintained a keen interest in those developments. The appendix of this article is devoted to those subsequent developments and how Weidmann himself might have reacted. Both of us have such good recollections of our real-life conversations with Silvio Weidmann that we decided we could achieve a better feel for the history and issues by using a dialogue format. We hope that, in that way, we may transmit the character of Silvio Weidmann better for those readers who will not have known him personally.

## Figures and Tables

**Figure 4.**
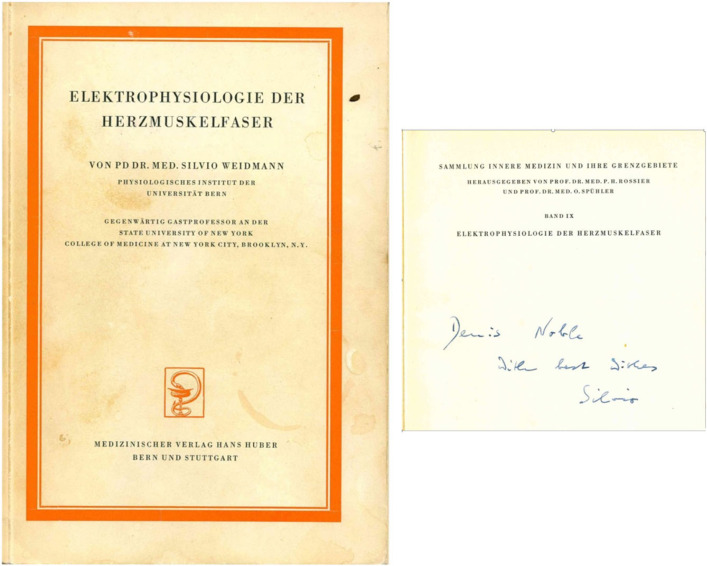
Denis Noble's copy of *Elektrophysiologie der Herzmuskelfaser* (1956) [5] signed by Silvio Weidmann. (Online version in colour.)

**Figure 5.**
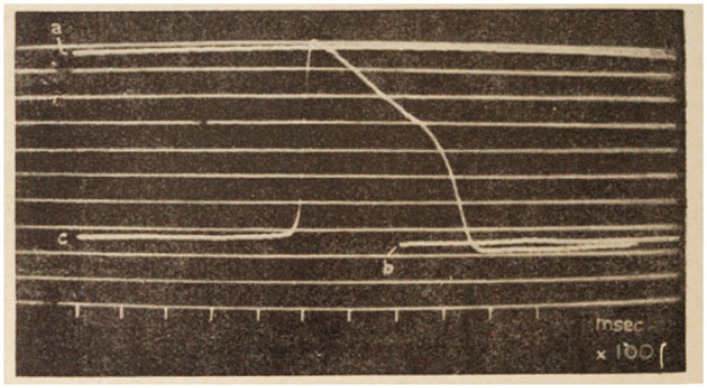
First recording of activity in a Purkinje fibre, from Coraboeuf & Weidmann (1949) [1]. Note that the action potential does not show a normal spike-like overshoot, as noted by the authors: ‘Il n'a pas été possible jusqu'à présent d'observer d'«overshoots» du potentiel d'action au-dessus du potentiel zéro plus importants que celui de la figure 1’. It is possible that the input to the amplifier had too low resistance. All the later recordings in Weidmann's papers clearly showed an overshoot.

**Figure 6.**
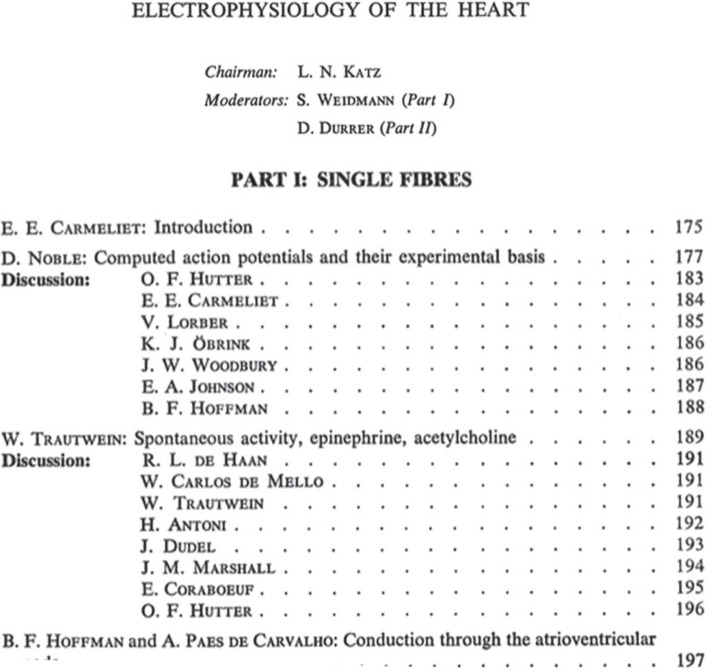
From the programme of the International Union of Physiological Sciences (IUPS) 1962 Congress in Leiden, Holland.

**Figure 7.**
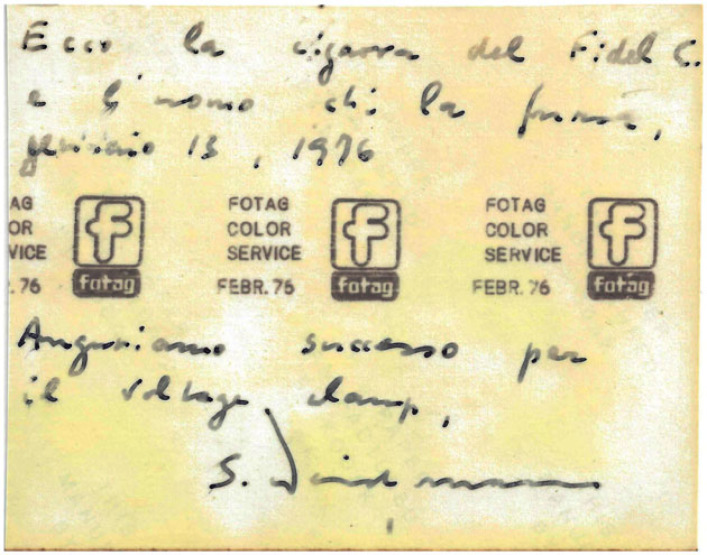
Back of a photograph of D.D. taken and autographed by Silvio Weidmann on 13 January 1976. The last sentence reads: 'Auguriamo successo per il voltage clamp*'.*

**Figure 8.**
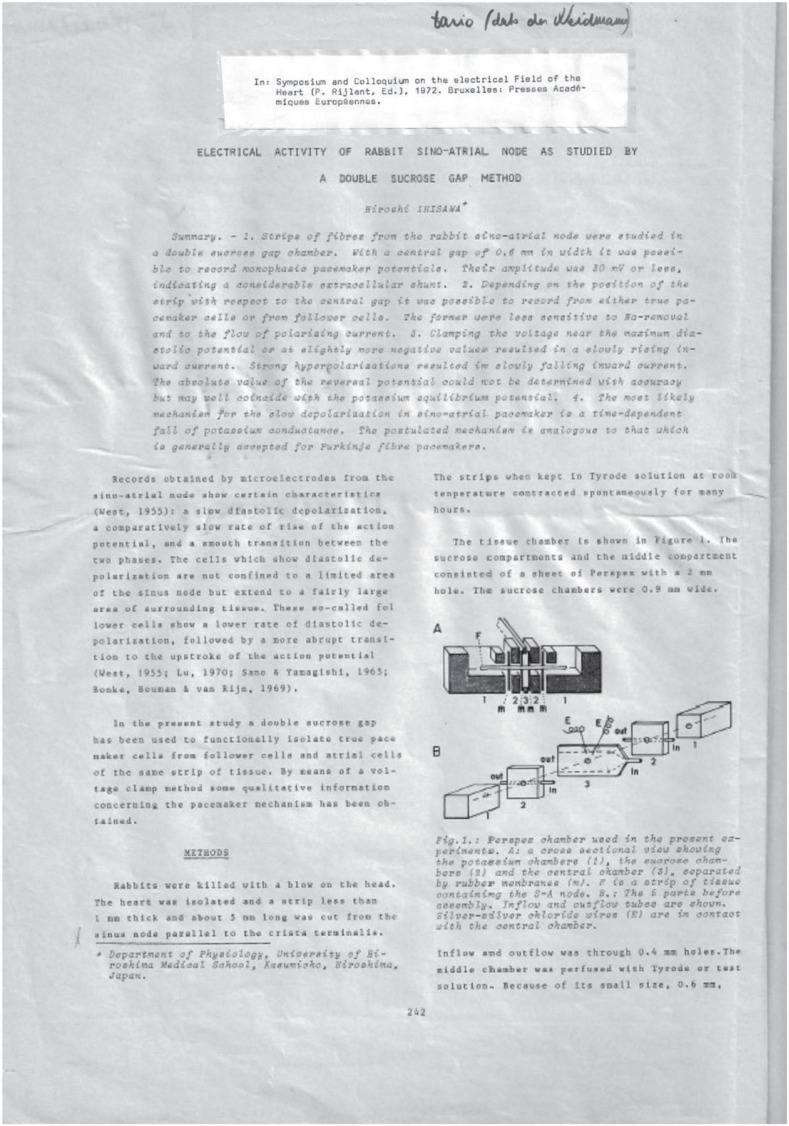
Abstract of the communication ‘Electrical activity of rabbit sino-atrial node as studied by a double sucrose gap method' delivered by Hiroshi Irisawa at a symposium in Brussels in 1972 [14]. Silvio Weidmann gave it to D.D. during his visit to Bern in January 1976.

## Data Availability

This article has no additional data.

## Appendix. ‘Dear Silvio Weidmann': a one-act play

**A.1. Introduction**

***…The play's the thing/Wherein I'll catch the conscience of the king [William Shakespeare, Hamlet II.2.557–8]***

*What follows is an imagined dialogue between the authors and Silvio Weidmann himself, in the format of a one-act play. It contains only our thoughts and reflections over his pioneering results and the way they have affected the development of cardiac electrophysiology in Europe and in the world. All the sentences attributed to Weidmann, except personal reminiscences and sentences extracted from published material, simply reflect our imagination. But they correspond to what we both knew of the man himself. Where possible, the texts reflect that knowledge*.

*This article is written as a series of questions/answers recounting the story of Weidmann's early experiments and what has developed from them*.

*Based on our personal relationships with Silvio Weidmann, we assume in this article that the personage we describe has an inquisitive character, doubtful about the new discoveries, but never hostile. In our story, Silvio Weidmann is imagined to be smart enough to digest them in one afternoon only*.

**A.2. A visit to Bern**

**(a) Setting the scene**

*Silvio Weidmann is still alive. Denis and Dario decide to go and visit him in Bern, and write him a letter*.

‘*Dear Silvio Weidmann, we should like to visit you and have your views on the developments in cardiac electrophysiology that followed your early findings.' Silvio promptly answers in a positive way, so the visit is organized*.

**(b) Action**

*It was a cold early afternoon of a winter day when Denis and Dario met at the Bern railway station. A taxi took them to Silvio's house, a large villa in a pleasant neighbourhood of Bern*.

*Silvio welcomed the visitors with his customary charm, inquiring about common acquaintances and family, always putting his guests at ease and making them feel most highly esteemed colleagues. His calm attitude, one of his most typical aspects, fitted well the character of an aged, celebrated Swiss academic. Still, his mind was clear and quick as ever*.

‘How are you doing?', *said Denis, and Dario added* ‘How are you today, Professor?' *Unlike Denis, Dario did not feel he deserved too much confidence with this great figure*.

‘I am very well.' *said Silvio*, ‘Please sit down.' *and showed them towards comfortable armchairs by the fireplace, where on a small table, hot chocolates with cookies were ready to warm up the discussion*.

*Denis broke the ice*:

‘Even as winter arrives, Silvio, your garden is lovely. You always told me you planted only what made it easy to maintain.'^1^

*Silvio was pleasantly surprised:*

‘Ah, you remember that! We Swiss have a different approach to gardening. It is the mountain life, you see. I recall Harald Reuter, years after his discoveries of calcium currents and the sodium–calcium exchange, taking up English-style gardening. Ewald Weibel told me of his amazement at the collection of fine lawn-cutting instruments that Harald and Liselotte had accumulated.^2^ Ewald, of course, with his grand mountain view and his garden on a slope, would never have trimmed his grass like that. Yes, just like my work in the laboratory, I like to keep things simple.'

*Denis recalled*:

‘That is what struck me when, as a student in Otto Hutter's laboratory at University College London, I read your monograph *Elektrophysiologie der Herzmuskelfaser* (figure 4). Even through the German language, which I have never mastered, I could appreciate your careful use of the early methods of glass microelectrode experiments. You had sent me a signed copy via Otto. It became my bible while I was doing the experiments with Otto on the potassium currents.'

*Silvio nodded*:

‘I was lucky in the period leading up to that monograph. Edouard Coraboeuf and I, in 1951, did the first glass microelectrode experiments on Purkinje fibres (figure 5), and then I was able to go to Cambridge to Alan Hodgkin's laboratory, where the Hodgkin–Huxley voltage-clamp experiments were being done, and where Andrew [Huxley] was working on their meticulous mathematical model of the nerve impulse. I was able first to learn by working on *Sepia* axons, and then to use current pulses myself to characterize heart muscle conductance properties.'

‘Yes,' *said Denis* 'those experiments became my bible again when I began the mathematical work leading to the 1960/62 modelling. I set myself the task of reproducing absolutely every one of the figures in your monograph.'

‘So you did' *agreed Silvio*.

‘When I moderated the 1962 IUPS Congress Symposium in Leiden, Holland, I was asking myself how you could possibly describe all of that in the 20 min you were allocated for your talk. You spoke so fast that I had difficulty following you! But we needed you to finish in time because seven other people were waiting to have their say in the discussion.' (figure 6).^3^

*Denis remembered*:

‘I wanted you to know that everything in your monograph could be reproduced. Alan Hodgkin became my thesis examiner just the year before, 1961. I found him very approachable.'

‘He was' *said Silvio*, ‘very much so for such a great scientist. He helped me whenever I needed the mathematics to understand my results. Like my experiments, I liked to keep the maths simple.'^4^

*Denis confirmed*:

‘Just like Otto. He told me when I first started using the massive 2 tonne Ferranti Mercury Computer in London that, from here on, I was "on my own". He was somewhat astonished when six months later I showed him the first reconstructions of your experiments. His comment was "Well, you have got your thesis. But it had better be right, because I am planning for Alan Hodgkin to be your examiner.' There was a slightly ominous twinkle in his eye. He knew I would be shaking somewhat in my shoes. My only consolation was that Otto was also shaking!'

*There was a pause of a few seconds, and Dario realized it was time for him to add his comments. He began with some hesitation, appropriate in the presence of this great figure:*

‘You may remember, Professor, that my first ever scientific visit was actually to your lab in Bern in 1976. You and my early mentor Arnaldo Ferroni knew each other, and on Ferroni's request, you had kindly agreed to have me visit. It was my great opportunity! In fact, it was also my first seminar, even if an informal one. I remember I was absolutely terrified to talk in front of Silvio Weidmann and Harald Reuter, who was also present!' ^5^

*Silvio nodded slightly:*

‘Yes I remember, I was in good terms with your head Arnaldo Ferroni, and I knew he was trying to set up a new team. In his letter, he introduced you as a bright and determined young researcher, and I liked the idea of helping him build a new electrophysiology lab in Milano, working on voltage clamp!'

*Dario felt quite gratified. Weidmann had been kind to him, and he now thought that he could return some of that kindness:*

‘I have a strong memory of that first visit, Professor. You were kind to me; you even invited me to your house, where I met your wife. The dinner was very pleasant; we chatted a lot. You had just been at a reception at the Cuban Embassy and had been given some cigars, the same brand smoked by Fidel Castro. This raised your hilarity, and you handed one of those cigars to me as a memento; you also took a picture and added on the back your wishes for success in the voltage clamp (figure 7).'

*Silvio listened attentively, and Dario felt he could add more recollections:*

‘But more importantly, that journey was sort of prophetic to me. I had worked briefly on spontaneously active chick embryonic cardiac cells (data later published [13]), and in my seminar I expressed an interest in the cardiac pacemaker. You were sensitive to that interest, and gave me a reprint of a communication to a 1972 "Symposium and Colloquium on the Electrical Field of the Heart" by Hiroshi Irisawa, who had previously visited Bern to run experiments on the double sucrose gap voltage clamp of amphibian sinus venosus (figure 8). I treasured that paper, which strongly affected my later choices. So, the real first ‘push' towards cardiac pacemaking I had received had come from Silvio Weidmann himself!'

*Silvio smiled, and Dario continued:*

‘I also remember, Professor, that you revealed a sort of deep, premonitory sensitivity. Seeing that I was deep in thought, you had asked me if there was anything worrying me, and anticipating my response you had said "Oh I know, forthcoming events!" Well, you were absolutely right. A few months later, I moved to Cambridge first and then to Oxford, and my career took off!'

*Silvio smiled openly now:*

‘I am glad if I have contributed to your career. After all, this was the aim of your visit!'

**A.3. The Na current (action potential generation, overshoot, propagation)**

*Denis picked up the subject, realizing it was time to get into more detailed scientific questions:*

‘Well, the outcome of those moves, Dario, is that you ended up in the Oxford laboratory. So, both of us have a lot to thank Silvio for. The Oxford laboratory was never the same again! And while we are on this subject of advice to young people, I want to express something I think all cardiac electrophysiologists feel, which is that you, Silvio, have inspired the field, not just with your pioneering work, but also with your personality. You are, without question, the father of our field, but you also filled that role with a quiet gentlemanly presence that we have all admired.’

*Silvio smiled with his characteristic quizzical look. Denis immediately switched back to the science, sensing that Silvio was just a little embarrassed:*

‘*…* So, I would like to take you back to 1949. It may be hard for young scientists today to imagine, but there were some very fundamental questions to be answered when it first became possible to record intracellular potentials directly with a microelectrode rather than using suction electrodes, which depended on accessing the inside potential by literally damaging an area where the suction electrode was applied. The question that was still in the air was whether, during electrical activity, the membrane simply depolarizes, in the way that would happen if there was a *general* increase in membrane permeability, or whether the depolarization is due to a *specific* ion permeability change. Hodgkin & Katz had answered this question in their 1949 paper showing the overshoot of the squid nerve action potential and its dependence specifically on sodium ions [15]. In your 1951 paper with Draper, you also answered that question unambiguously for the heart by showing that the peak overshoot of the cardiac action potential behaves as a sodium electrode [2].'

*Silvio agreed:*

‘Yes. You can see the evidence for the old conventions from the suction electrode work in the fact that we were still using the outside potential to describe the directions of change. The overshoot was described as *Aussen negativ* (outside negative), while the resting potential was labelled as *Aussen positiv*.'

*Denis continued*:

‘That was also true when I first computed the Purkinje fibre action potentials using my 1960 modification of the Hodgkin–Huxley equations [6]. In the computer program all the potentials were referred to the outside! When I drew the diagrams for publication, I had to turn everything around. That led to a problem when Alan Hodgkin examined my thesis. Some of the arrows in the circuit diagrams had to be inked out and reversed. Those are just matters of definition. What is not just a matter of definition is the fact that your results made it easy to formulate equations for the sodium channel closing (inactivation) gate (*h*). You used the action potential upstroke velocity to estimate the magnitude of the sodium current when depolarizing from various potentials. Given the impossibility at that time of a voltage clamp technique, that was an ingenious way to get the inactivation curve. But it nevertheless left me with the problem of getting suitable equations for the activation gate (*m*). I tried the original Hodgkin–Huxley formulation, but that did not work. So I knew that either the activation process was different in the heart, or there must be other currents involved. I chose the first option. It would have been mere speculation in 1960 to conclude that there was another ion current channel. That came later in 1967 with Harald Reuter's discovery of calcium channels [8].'

*Silvio*: ‘For me, that was always a difficult part of your 1962 paper.'

*Denis couldn't agree more*:

‘You were not alone, Silvio. Alan Hodgkin was sufficiently concerned about it that he suggested I should leave one of the relevant diagrams out of my 1962 *Journal of Physiology* article (fig. 9 of [7]). I am pleased that I resisted his advice. From a historical point of view, it is important that people should know that the evidence for other inward currents was always there. That makes it easier to understand why the model worked so well, and reproduced all your current pulse experiments. Those modifications of the *m* equations enabled the model to do that. Of course, today we would not need to modify the activation equations in the same way. The calcium, and sodium–calcium exchange currents perform the relevant roles in generating the plateau of the action potential. And there are persistent sodium currents too.'

*Silvio had a question*:

‘I am glad you explained that. But why do you think that everything had to become so complicated? Your 1960/62 formulation worked so well.'

*Denis' answer was swift:*

‘It did, Silvio. Which is why in my subsequent work, I took great care to map old and new models onto each other. That approach applied also to mapping the old *I*_K2_ model and the new *I*_f_ model, but here is where I hand back to Dario.

*Denis immediately remembered something else he needed to add:*

‘Ah, wait a minute. Just before doing so, I do have another remark on this problem. It is not enough in science to develop new theories, we need always to understand how theories map onto each other. The physicists know this very well, which is why they take great care. Einstein's relativity equations explain Newton's work perfectly well, and the mapping also enables us to know in what range of parameters the old model can still be used. As to why there are so many ionic currents, I suspect the answer to that question lies in the evolution of robustness in biological processes.'

**A.4. The diastolic depolarization (conductance measurements, *G*_K_-decay theory, funny current)**

*Denis's last sentences were an implicit invitation to Dario. Dario's approach was timid and respectful:*

‘Professor, in your 1951 experiments in Purkinje fibres, you applied small repetitive current steps to investigate conductance changes during the action potential [4]. It was a clever protocol that generated plenty of new insight into the mechanisms responsible for the electrical activity of cardiomyocytes. The data provided were very influential for a long time. The protocol allowed to identify changes occurring during the upstroke, during the plateau, during repolarization …. Perhaps the only problem concerned the diastolic depolarization …,'

*Silvio's eyebrows raised almost unperceptively. Dario continued:*

‘… where conductance changes led you to state that "during diastole the slow depolarization is associated with an increase in membrane resistance" [4, p. 228]. This was a solid result, later fully confirmed for example by Trautwein's laboratory [16], that affected heavily later studies, but as you know, although perfectly valid, it was in a sense misleading.'

*Silvio's eyebrows raised a little more. Dario did not want to create embarrassment, so he moved on quickly to get to the question he wanted to ask:*

‘You are probably aware that later analysis, based on your original observations, led to the proposal that the diastolic depolarization is due to a decaying outward current, the so-called *G*_K_-decay hypothesis— a proposal that was met with great success, confirmed by several studies. However, this proposal had to confront a formidable twist in the late 1970s, when a new mechanism was described in true pacemaker cells, the so-called "funny' current", inward and activating at diastolic voltages [9]. Along with many other consequences, this finding also led to a re-interpretation of pacemaking in Purkinje fibres, which in fact turned the original hypothesis upside-down [10,11]. How did you react to these new developments?'

*Silvio addressed his guest with an enigmatic smile, impossible to decipher if hinting to a little sarcasm, but answered with his typical gentle disposition:*

‘Yes, I have heard about your "funny" current, I have been reading some literature on the subject. It has given rise to some discussion with my colleagues, especially with Mario Vassalle. You see, even before I ran the 1951 experiments, a decrease of membrane conductance was very much expected for the diastolic depolarization. Do you remember the time course of conductance in the Hodgkin–Huxley model of giant axon action potential?'

*Dario was slightly taken aback:*

‘Ehmmm, I think so, yes.'

*Silvio continued:*

‘Well, the slow depolarizing phase following the action potential, the so-called "refractory period", is due to the decline of the K^+^ conductance. So I thought it was reasonable to measure a conductance decrement during the Purkinje fibre's diastolic depolarization. But I do not agree that the conductance measurement during diastolic depolarization was a "misleading" observation.'

*Silvio's mood was always gentle, but it had become firmer:*

‘When Mario Vassalle, working in Bern, published in 1966 the first recordings of voltage-clamp steps at diastolic voltages, he saw, as he himself stated in the paper, "an inward current which progressively increased with time" [17, p. 1336]. Would it have been more intuitive simply to declare it an inward increasing current? Maybe, but my early experiments demonstrating a decreasing conductance made a control necessary. So Mario used the same technique, this time applied during voltage clamp. And you know what the result was.’

*Silvio looked intensely at Dario, who was ready to respond; he had read carefully Vassalle's paper:*

‘Yes I know, he measured a conductance decrease. And this obviously supported a "diminution of *G*_K_ as the cause of the progressively increasing inward current", since "a time-dependent increase of G_Na_ would have required an opposite finding" to use Vassalle's words.' [17, p. 1338].

*Silvio picked up again:*

‘So you see what I mean, rather than misleading, we can speak of an unfortunate suggestion. A correct measurement incorrectly read, and leading to a wrong interpretation. Many years have gone since those experiments were run, and nobody could have known at the time that, along with the activation of the inward "funny" current, also a depletion of extracellular K^+^ ions occurs when applying hyperpolarizing steps, which strongly affects the time course of conductance. And the conductance measurement wasn't the only ‘unfortunate' finding. Stepping to more and more negative voltages, Mario found that the direction of current change reversed, an indication of a K^+^ current.'

*Dario was caught off-guard:*

‘But …'

*Silvio did not budge:*

‘Oh I know, Mario's experiments were run in a Na^+^-free solution, so there could not be any Na^+^ contribution anyway. But all in all, the entire set of data converged to indicate that the diastolic depolarization of Purkinje fibres is due to a fall in potassium conductance. Mario and I discussed those data. We were obviously aware that an inward current had to be there to drive the diastolic phase—a depolarization requires a net inward ionic current—so we assumed the decay of *G*_K_ simply unmasked a background Na^+^ current still to be identified. But as a whole, the scenario appeared quite reasonable. And …’

*Silvio's gaze now turned to Denis, who was listening attentively:*

‘… we felt much more confident when 2 years later, Denis and Dick Tsien described the *I*_K2_ current [18]. It was a full confirmation of our data! Mario was slightly disappointed; he thought he could have extracted more information from his 1966 data, but still the description of the *I*_K2_ current was an elegant validation of his findings, and confirmed the *G*_K_-decay hypothesis as the main mechanism underlying the diastolic depolarization.'

*Silvio smiled and his gaze returned to Dario:*

‘And in their work, clear current reversal records were reported also in the presence of external Na^+^… . Anyway, to come to your question about the "funny" current, your work with Hilary Brown and Susan Noble was published in 1979 [9] , more than a decade later, and it was in the sino-atrial node, not in Purkinje fibres, so it looked like an interesting finding but not a disturbing one. But when you published the two re-interpretation papers in the *Journal of Physiology* in 1981 [10,11], I admit it was disturbing. Mario did not take it lightly. He was incredulous. He could not accept that a set of experimental conditions essentially due to an unpredictable circumstance, such as the depletion of K^+^ ions in the extracellular clefts of Purkinje fibres, could lead to mistaking an inward increasing current for an outward decreasing one!’

*Silvio stopped, and Dario thought it was time to step in:*

‘I know what you mean, Vassalle wrote me several letters discussing the issue with complex arguments.^6^ Those findings left many incredulous. Anyway, this part of the story has been debated at length in many reviews. You yourself, Professor, while mentioning the limitations of the voltage-clamp technique of Purkinje fibres with two microelectrodes, noted in one of your reviews [19] that among other technical flaws "accumulation/depletion of ions in the interspace may seriously affect interpretations, and too often it would be difficult to distinguish between an inward current decreasing and an outward current increasing with time"!

On the other hand, considering the conductance measurements made during the diastolic depolarization, you know that in fact your old data are perfectly valid and fully congruent with the new interpretation!'

*Dario's gaze turned to Denis, who nodded in agreement:*

‘When in January 1981 I told Denis over the phone (a phone call mentioned in a few review publications) that the *I*_K2_ current simply did not exist and had to be replaced by the newly discovered sino-atrial "funny" current, and that the decay of *I*_K2_ was to be replaced by the activation of *I*_f_ during diastole, Denis was immediately aware of the revolutionary content of that finding and realized that it was necessary to check the consistency of the new concept with known experimental data. He and I worked on a new numerical model, which was published in 1985 [12], where the electrical activity of Purkinje fibres was reconstructed. The model reproduced faithfully the rhythmic activity of Purkinje fibres, in the assumption that pacemaking was triggered by activation of an inward, not deactivation of an outward current. Yet, your conductance measurements could also be safely reproduced, despite the radical re-interpretation of *I*_K2_!'

*Silvio was listening carefully and had become more thoughtful. He stepped in:*

‘Since you mentioned the conductance measurements, I have thought about it. It looks to me that the real question was not so much the conductance change itself, but its relevance to the time course of voltage. It was in a sense a conceptual flaw.'

*Dario scratched his head, and Silvio continued:*

‘Changes in the membrane conductance do not give unequivocal evidence for the specific changes in the ionic currents generating them. So for example at diastolic voltages a K^+^ component can give a large contribution to membrane conductance, but a little contribution to current flow, since voltage is close to the K^+^ reversal potential, while the opposite occurs for a component with a more depolarized reversal potential, such as a Na^+^ or a mixed cation component. What is important in terms of driving the diastolic depolarization and determining its rate is not the time course of membrane conductance, but the net ionic current. And a depolarization requires an inward current, so an inward current was what to look for. It is not surprising to have a decrease of membrane conductance, as predicted by the *G*_K_-decay model, and at the same time an activating inward current as the triggering mechanism!'

*Denis and Dario were not surprised by this clear-cut, open-minded analysis. But Dario was eager to ask a question:*

‘What I should like to ask you is, do you think that all these developments, even those that apparently conflicted with your findings, are the natural evolution of your work? Do you think that young researchers in cardiac electrophysiology working today can be all considered as disciples of yours, all mentees following your pioneering steps?'

*Silvio smiled:*

‘I do not know, this is a far-fetched assumption, I wonder if you are trying to flatter me?’

*Dario was half surprised, but then realized Silvio was joking. Silvio continued:*

‘No, I'm joking, I know it is a serious question. As I say, it is a far-fetched assumption, but at the same time it is difficult not to see a continuity in the developments of cardiac electrophysiology based on the data I collected so long ago. So in a sense, yes, I like the idea of being seen as one of the "‘fathers" of cardiac electrophysiology!'

**A.5. Conclusion**

*Silvio's distinctive bonhomie had never faded during the visit, but when Denis and Dario heard him accepting such a praising compliment, they also perceived a hint of tiredness in his voice. A few long moments passed in silence, a sign that the visit had come to an end. Silvio accompanied his guests to the door, and bade them farewell warmly*.

‘We have had a good chat.'*, he said appreciatively.* ‘Thank you for coming.'

*Days are short in Bern in the winter, and it was dark when Denis and Dario walked toward the waiting taxi. Turning to wave a last good bye, they saw Silvio waving his hand on the door.* ‘And come again!', *he said. Denis and Dario watched each other and smiled. They were both thinking the same thing:*

‘What a scientist and what a man! I feel indebted to Silvio, my career has been so strongly affected by his work. But in fact, how many cardiologists and cardiac physiologists have shaped their knowledge on the basis of Silvio Weidmann's results? If it wasn't for Silvio Weidmann, what would there be of cardiac electrophysiology in our days?'

*Denis and Dario knew perfectly well they were having the same thoughts. But the taxi was speeding in the Bern night, and neither spoke*.
